# Phenotypic plasticity, canalisation and developmental stability of *Triatoma infestans* wings: effects of a sublethal application of a pyrethroid insecticide

**DOI:** 10.1186/s13071-021-04857-5

**Published:** 2021-07-06

**Authors:** Julieta Nattero, Gastón Mougabure-Cueto, Vincent Debat, Ricardo E. Gürtler

**Affiliations:** 1 Departamento de Ecología, Genética y Evolución/Laboratorio de Eco-Epidemiología, Ciudad Autónoma de Buenos Aires, Facultad de Ciencias Exactas y Naturales, Universidad de Buenos Aires, Buenos Aires, Argentina; 2 Instituto de Ecología, Genética y Evolución (CONICET-IEGEBA), CONICET–Universidad de Buenos Aires, Buenos Aires, Argentina; 3 Laboratorio de Investigación en Triatominos (LIT), Centro de Referencia de Vectores (CeReVe), Ministerio de Salud de La Nación, Santa María de Punilla, Córdoba, Argentina; 4 Consejo Nacional de Investigación Científicas y Técnicas (CONICET), Buenos Aires, Argentina; 5 Institut de Systématique, Evolution, Biodiversité (ISYEB) (UMR7205), CNMuséum National d’Histoire Naturelle, Sorbonne Université, Ecole Pratique des Hautes Etudes (EPHE) and Université des Antilles, Paris, France

**Keywords:** Canalisation, Developmental instability, Phenotypic plasticity, Pyrethroid, Sublethal doses, Vector control, Wing size, Wing shape

## Abstract

**Background:**

Triatomine control campaigns have traditionally consisted of spraying the inside of houses with pyrethroid insecticides. However, exposure to sublethal insecticide doses after the initial application is a common occurrence and may have phenotypic consequences for survivors. Here, using *Triatoma infestans* (the main vector of Chagas disease in the Southern Cone of South America) as a model species, we quantified the effects of exposure to a sublethal dose of pyrethroid insecticide on wing morphology. We tested if the treatment (i) induced a plastic effect (change in the character mean); (ii) altered environmental canalisation (higher individual variation within genotypes); (iii) altered genetic canalisation (higher variation among genotypes); and (iv) altered developmental stability (higher fluctuating asymmetry [FA]).

**Methods:**

Each of 25 full-sib families known to be susceptible to pyrethroid insecticides were split in two groups: one to be treated with a sublethal dose of deltamethrin (insecticide-treated group) and the other to be treated with pure acetone (control group). Wings of the emerging adults were used in a landmark-based geometric morphometry analysis to extract size and shape measurements. Average differences among treatments were measured. Levels of variation among families, among individuals within families and among sides within individuals were computed and compared among treatments.

**Results:**

Wing size and shape were affected by a sublethal dose of deltamethrin. The treated insects had larger wings and a more variable wing size and shape than control insects. For both wing size and shape, genetic variation was higher in treated individuals. Individual variations and variations in FA were also greater in deltamethrin-treated insects than in control ones for all full-sib families; however, the patterns of shape variation associated with genetic variation, individual variation and FA were different.

**Conclusions:**

Insects exposed to a sublethal dose of deltamethrin presented larger, less symmetrical and less canalised wings. The insecticide treatment jointly impaired developmental stability and genetic and environmental canalisation. The divergent patterns of shape variation suggest that the related developmental buffering processes differed at least partially. The morphological modifications induced by a single sublethal exposure to pyrethroids early in life may impinge on subsequent flight performance and consequently affect the dynamics of house invasion and reinfestation, and the effectiveness of triatomine control operations.

**Graphical Abstract:**

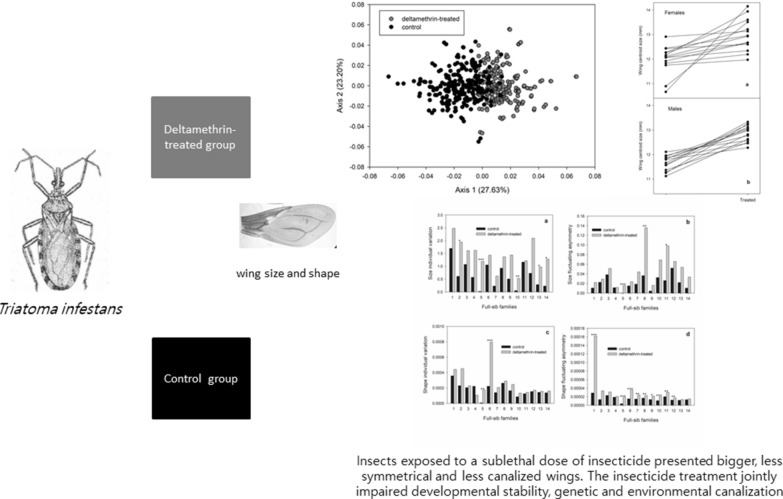

## Background

*Triatoma infestans* (Hemiptera, Reduviidae, Triatominae), the main vector of *Trypanosoma cruzi* in the Southern Cone of South America [1], is showing an extreme trend in the process of domestication [2, 3]. House spraying with pyrethroid insecticides has been the traditional method to prevent human infection with *T. cruzi* in Latin America since the mid-1980s [4–6]. The suite of insecticide control campaigns that were carried out reduced the species’ distribution range and house infestation levels, which in turn has lead to the interruption of parasite transmission in several regions since the 1990s. However, *T. infestans* populations have persisted in the Gran Chaco ecoregion of Argentina, Bolivia and Paraguay despite recurrent vector control campaigns [1, 6, 7]. Vector persistence is associated with poor housing conditions, technical shortcomings during insecticide application, active triatomine dispersal from residual or untreated foci and/or the emergence of pyrethroid resistance from the late 1990s onward [8–13].

Phenotypic variation and the factors that shape this variation are fundamental aspects of evolutionary biology [14] because they are directly related to the evolutionary potential of populations and adaptability. The three major processes involved in phenotypic variability are plasticity, canalisation and developmental stability [15]. According to current definitions of these concepts, plasticity is the ability of genotypes to produce more than one phenotype when exposed to different environments. Canalisation is the ability to produce a consistent phenotype despite genetic and environmental influences. Developmental stability is the ability to produce a consistent phenotype despite random developmental errors (e.g. [16]). The relationship between the processes involved in developmental stability, canalisation and plasticity has long been debated [15–21]: are these buffering mechanisms different, or do they mostly correspond to aspects of the same process?

The sources of phenotypic variation (stochastic, environmental and genetic) and developmental buffering processes may be estimated through an experimental design that includes different genetic families replicated in different environments. Phenotypic variation can be estimated experimentally at four levels: (i) at the lowest level, within a genotype and an environment, variation within individuals is generally measured by fluctuating asymmetry (FA) as the variance of the difference between right and left values of bilateral traits whereby FA is assumed to reflect developmental noise and thus estimates developmental stability [22]; (ii) variation among individuals within a genetic family and within an environment mostly originates from micro-environmental variation and thus reflects micro-environmental canalisation; (iii) variation among families within an environment mostly originates from genetic variation and thus reflects genetic canalisation; and (iv) macro-environmental variation (phenotypic plasticity) can be measured as variation among families placed in different environmental conditions.

*Triatoma infestans* displays plasticity in various traits in response to different environmental conditions [23–25]. Insect wings are involved in dispersive flights, which imply ecological, evolutionary and epidemiological consequences. In *T. infestans*, flight is important for house invasion and reinfestation following insecticide spraying or other control actions. Wing shape, as assessed by geometric morphometrics, may have an important impact on flight (e.g. [26]). Wing shape was found to be a useful phenotypic marker of the relationship between FA and pyrethroid exposure in a rural area where *T. infestans* persisted after pyrethroid applications [27].

In other insect species, sublethal pyrethroid exposure caused conspicuous changes in the motor activity of the beetle *Platynus assimilis* [28], changes in biological parameters and behavioural responses to host cues in the egg parasitoid *Telenomus busseolae* [29] and a locomotor deficit in the honeybee *Apis mellifera* [30]. Sublethal doses of insecticide causing resistance in an insect population have been demonstrated in laboratory selection programmes. These programmes typically applied sublethal insecticide doses that allowed survivors in each generation and the accumulation of many mutations over several generations; the latter resulted in a polygenically inherited resistance [31]. Insecticide-based selection may exert pleiotropic effects, including phenotypic modifications such as reduced life expectancy, changes in behaviour or changes in level of the FA of morphological traits [e.g. 32, 33]. *Triatoma infestans* nymphs displayed symptoms of poisoning, toxicity at different temperatures and hyperactivity after exposure to sublethal doses of deltamethrin [34–36]. Whether the sublethal doses of pyrethroid insecticides impact plasticity, canalisation and developmental stability has not been investigated for any Triatominae species. Research may cast light upon the phenotypic consequences of exposure to the toxicant. In the field, exposure of insects to sublethal insecticide doses usually occurs after the initial application, as these molecules are rapidly degraded by rainfall, temperature and sunlight [37]. Sublethal insecticide effects may exert biological, physiological, demographic and behavioural effects on individuals or populations and affect their fitness [37–39].

In the study reported here we examined the effects of a sublethal dose of pyrethroid insecticide applied to first-instar nymphs of *T. infestans* on variations in wing size and wing shape in the emerging adults. We hypothesised that, by altering the amount and patterns of phenotypic variation (including genetic variation), insecticide treatment may affect the potential for adaptive evolution of *T. infestans*. The aims of this work were to evaluate whether: (i) wings are a plastic trait susceptible to change when exposed to a sublethal dose of a pyrethroid insecticide; (ii) different full-sib families differ in wing size and shape (i.e. are genetically variable); (iii) FA, individual variation and genetic variation show higher levels in treated insects than in control insects; and (iv) the patterns of wing shape change involved in FA, individual variation and genetic variation among families are similar. A negative answer referring to similar variation would suggest that the processes involved in buffering of the diverse sources of variation are different (e.g. [17, 19] but see [16]).

## Methods

### Insects and deltamethrin susceptibility

*Triatoma infestans* populations used in the current study were collected during a cross-sectional survey of house infestation with triatomines in three neighbouring rural communities in Chaco Province, Argentina, in May 2014: Tacuruzal (Quitilipi and Maipú Departments), Pampa Bandera (25 de Mayo Department) and Pampa Esperanza (Maipú Department). Houses in this area had not been sprayed with pyrethroid insecticide for about 10 years before our survey. After insect collection, all houses positive for *T. infestans* were immediately treated with pyrethroid insecticide (β-cypermethrin at 50 mg/m^2^) by vector control personnel using standard procedures.

Eggs from *T. infestans* females collected during the cross-sectional survey were pooled by location and the emerging first-instar nymphs were tested for deltamethrin susceptibility at a reference laboratory (Centro de Investigaciones de Plagas e Insecticidas, Villa Martelli, Argentina). Deltamethrin susceptibility tests were carried out following a standardised protocol [40]; all populations were found to be fully susceptible to deltamethrin.

### Toxicological bioassay

A sublethal dose of deltamethrin (technical-grade 99% purity; Sigma-Aldrich, Buenos Aires, Argentina) was administrated to first-instar nymphs of *T. infestans* (5–7 days old, kept unfed after emergence) [40]. The dorsal abdomen of each insect was treated with 0.2 μl of a deltamethrin acetone solution (0.005 mg/ml), i.e. 1 ng per insect (the median lethal dose [LD_50_]), using a 10-μl Hamilton syringe equipped with a Hamilton PB-600–1 Repeating Dispenser (Hamilton Company, Reno, NV, USA). Insecticide doses lower than the LD_50_ are considered to be sublethal [37]. Two types of control groups were designated: insects treated topically with 0.2 μl of pure acetone, and untreated insects (see subsection Experimental design and data collection). Insect mortality was evaluated 24 h after exposure. Death was judged from the insect’s inability to walk from the center of a circular 7-cm-diameter filter paper [40].

### Experimental design and data collection

Insects collected in the three study locations were maintained in the Centro de Referencia de Vectores (CeReVe, Punilla, Argentina) as free-mating separate stocks at room temperature (approximately 24 °C) for three generations. One hundred fifth-instar nymphs from these mating stocks were transported to the insectary at FCEN-UBA where the experiments were carried out. Groups of ten fifth-instar nymphs each were held in cylindrical vials kept at 26 ± 2 °C, 60–70% relative humidity and a photoperiod of 12:12 h (light:dark) throughout the experiments. Nymphs were fed regularly with rabbit blood provided* via* an artificial feeder until adult emergence. Newly emerged adult triatomines from the same locality (1 male and 1 female) were held separately in cylindrical glass vials. We obtained 25 couples; the entire offspring of each couple was considered a full-sib family.

For each full-sib family, all emerging first-instar nymphs were divided haphazardly in three groups. Group 1 was treated topically with a sublethal dose of deltamethrin; group 2 was treated with pure acetone (first control group); and group 3 was not treated (second control group). A preliminary analysis showed that both wing size and shape did not differ significantly between control groups 2 and 3. Since group 3 was not well represented in all full-sib families, only group 2 was included in subsequent analyses. All full-sib families in which > 10 insects survived per treatment (hereafter, group 1: deltamethrin-treated group; group 2: control group) were included in this study. Of 352 first-instar nymphs, 172 (48%) survived the application of deltamethrin; 258 (95%) of 272 first-instar nymphs from the control group survived the application of acetone. All nymphs that survived the topical application were fed on rabbit blood every 15 days and kept under laboratory conditions until adult emergence, when they were killed and stored in 70% ethanol. Of the 25 full-sib families at the onset of experiments, we ended up with 14 full-sib families, each of which included 4–20 insects per sex per group (Table 1). The final sample size was 430 adult triatomines and 860 wings.

**Table 1:** Identification of the full-sib families of *Triatoma infestans* used in this study, including the number of females and males in the control and deltamethrin-treated groups

Full-sib family	Female (*n*)		Male (*n*)	
	Control sample size	Deltamethrin-treated sample size	Control sample size	Deltamethrin-treated sample size
1	11	4	8	4
2	6	5	4	10
3	18	4	18	6
4	9	5	4	4
5	9	5	4	6
6	7	10	19	11
7	4	8	10	8
8	13	6	4	10
9	4	10	15	6
10	16	6	7	4
11	10	6	6	4
12	9	13	6	5
13	12	4	6	7
14	6	6	8	4
Total	134	92	119	89

The left and right wings of these 430 insects were mounted between a microscope slide and cover slip and then photographed with a digital camera (model S9900; Nikon Corp., Tokyo, Japan) attached to a stereomicroscope (model Stemi SV-11; Carl Zeiss AG, Oberkochen, Germany‎) at 6× magnification. All images included a reference scale. We collected ten type-I landmarks positioned at vein intersection, as described in [41], using the tpsDig2 version 2.31 software programme (http://life.bio.sunysb.edu/morph/). Digitisations of both right and reflected left wings were repeated twice to estimate the measurement error (ME), which is of critical importance when analysing FA [42].

### Morphometric analysis and statistical treatment

Data on the wing shape was extracted with a generalised full Procrustes fit and a projection to shape tangent space [43]. A principal component analysis (PCA), based on the covariance matrix of landmark coordinates after the Procrustes fit [43], was used to examine the dimensionality of shape variation and to extract shape variables for subsequent analyses. We computed centroid size (CS; i.e. the square root of the sum of squared distances from each landmark to the centroid of the configuration) as a measure of wing size [43]. These steps of morphometric analysis were performed using MorphoJ version 1.07a software [44].

To quantify the effects of study factors (i.e. full-sib family and treatment: control and deltamethrin exposure) and their interaction, we used analyses of variance (ANOVAs) on wing CS and multivariate analyses of covariance (MANCOVAs) on wing shape variables. ANOVAs for mean wing size included the fixed effect of treatment. MANCOVAs on shape variables included full-sib family and treatment as main effects and CS as a covariate. Size reaction norms can be represented as a line linking the mean wing size of each full-sib family within the control and deltamethrin-treated groups. We additionally performed a canonical variate analysis (CVA), including full-sib family and treatment as clustering variables, to visualise whether the dataset was structured by treatment. Shape reaction norms can be represented as lines linking the position of each full-sib family within control and deltamethrin-treated groups [45].

The amount of individual and FA variation for wing size and shape was measured for each full-sib family and each group (control and treated) using regular ANOVAs for size and Procrustes ANOVAs for shape [46], considering individual, side, the interaction between individual and side as effects, the replicates being left in the residual term and accounting for ME. The ANOVA mean square (MS) related to the individual effect was used as an estimator of individual variation. The MS related to the interaction (individual × side) was used to compute the FA10 index as FA10 = (MS_interaction_ − MS_ME_)/2 following [42]. This FA index indeed accounts for ME and provides a reliable estimation of FA. Variance estimates were then compared among treatments for each full-sib family using standard F-tests. To test whether the most variable families under the control conditions also tended to be the most variable once treated, we used the Kendall tau correlation coefficient for individual variation and FA (computed with Wessa.net freely available at https://www.wessa.net/). In order to test the relationship between canalisation and developmental stability, we computed Kendall’s tau between individual variation and FA across full-sib families within each treatment. The effect of treatment on genetic variation for wing size and shape was assessed by measuring variation among full-sib families. For both the treatment and control groups, the MS in an ANOVA on wing size with full-sib family as a single effect was calculated; the corresponding Procrustes MS was used to measure shape variation across full-sib families.

To investigate whether full-sib family and treatment involved similar changes in landmark position (i.e. patterns of shape variation), we used the following approach. For each full-sib family and treatment, we computed two covariance matrices: one corresponding to differences among individuals (environmental canalisation) and one corresponding to FA (developmental stability), resulting in 56 matrices. Additionally, the genetic covariance matrix (genetic canalisation) was estimated across full-sib families within each treatment (2 matrices). We used a matrix correlation method to compare covariance matrices by pairs, including the diagonal elements of the matrices because both the variances and covariances can provide information on the similarity of covariance matrices [18]. Matrix correlations were tested with a matrix permutation test against the null hypothesis of complete dissimilarity of covariance structures [47]. Because the coordinates of each landmark are not independent, permutations were done on the landmarks and not on the individual coordinates in the covariance matrix [46].

To compare environmental canalisation, developmental stability and genetic canalisation matrices across families and treatments, we used a metric multidimensional scaling (e.g. [48]) as an ordination analysis [18, 19, 49]. This analysis (also known as principal coordinates analysis [PCO], which is related to PCA [50]) uses a distance matrix between all possible pairs of covariance matrices as input data. We used (1 − [correlation between the two matrices]^2^) as a distance metric [18, 19]. The ordination of PCO helps to visualize the relationship between covariance matrices; the closer two matrices are in the PCO ordination, the more they are correlated and the more similar the patterns of landmark covariation. We first applied a PCO simultaneously to all 58 matrices (individual variation and FA matrices for all families under the two conditions + the 2 genetic covariance matrices). This procedure allowed us to capture in a single step the overall similarity of the effects of micro-environmental variation, developmental noise and genetic variation. To further study if individual and FA variation reacted similarly to the treatment, we then ran PCO on the corresponding matrices alone. Since there were 14 families, the genetic matrices have a maximum of 14 dimensions. We thus reduced the dimensionality of all covariance matrices to 14. These analyses were conducted using R running on RStudio version 1.2.5019 (2009–2019; RStudio Inc., Boston, MA, USA). For all analyses, sexes were pooled to increase statistical power when differences between sexes were not detectable.

## Results

### Deltamethrin-treated individuals exhibited bigger and more variable wings with different mean wing shape

**Fig. 1:**
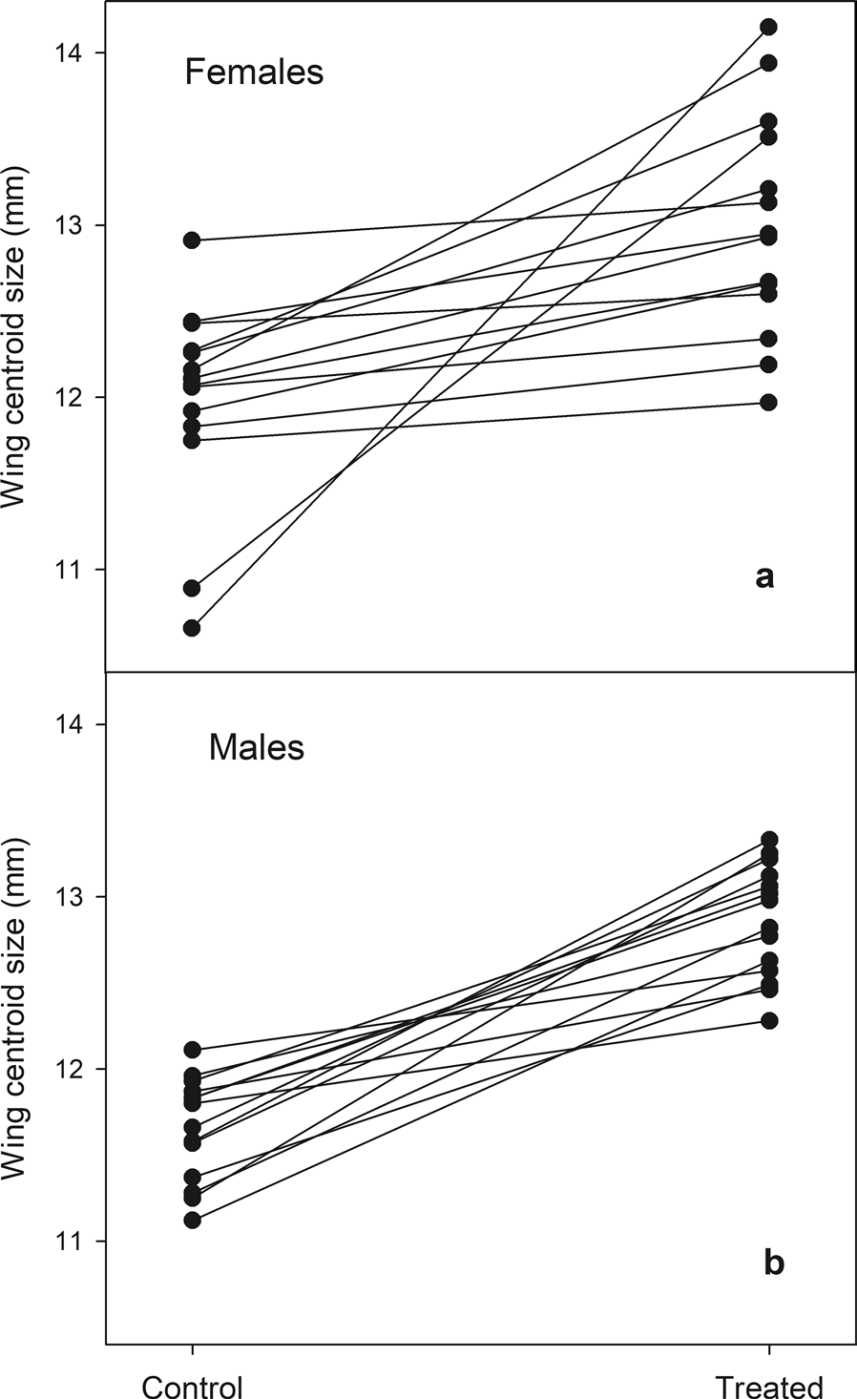
Wing size reaction norm for female (**a**) and male (**b**) *Triatoma infestans*. Wing size is measured as centroid size. Connecting lines represent the direction of size change due to treatment within a full-sib family, i.e. size reaction norms

Wing size and shape were affected by a sublethal dose of deltamethrin and varied across full-sib families. Both female and male adults that had been treated with deltamethrin had larger and more variable mean wing CS (Table 2). The full sib-family effect from the ANOVA also modified mean wing size, indicating the presence of genetic variation for wing size. A strong interaction effect between treatment and full-sib family was detected both in females and males, indicating that deltamethrin effects on wing size differ across full-sib families (Table 2). In other words, there is genetic variation for size plasticity. Figure 1 shows that although some lines cross each other, most reaction norms are nevertheless roughly parallel, especially in males. Figure 1 illustrates that the increase in size is a global common effect of the exposure of wings to deltamethrin.

**Table 2:** Results from analyses of variance on wing size (centroid size) for control and deltamethrin-treated conditions, full-sib families and the interaction term (treatment × full-sib family)

Wing size	Effect	*df*	SS	MS	*F*	*P*-value
Female	Treatment	1	47.62	47.62	274.25	< 0.0001
	Full-sib family	13	24.80	1.91	10.99	< 0.0001
	Treatment × full-sib family	13	38.22	2.94	16.93	< 0.0001
	Residuals	199	34.55	0.17		
Male	Treatment	1	60.60	60.60	333.26	< 0.0001
	Full-sib family	13	6.69	0.51	2.83	0.001
	Treatment × full-sib family	13	10.66	0.82	4.51	< 0.0001
	Residuals	180	32.73	0.18

**Fig. 2:**
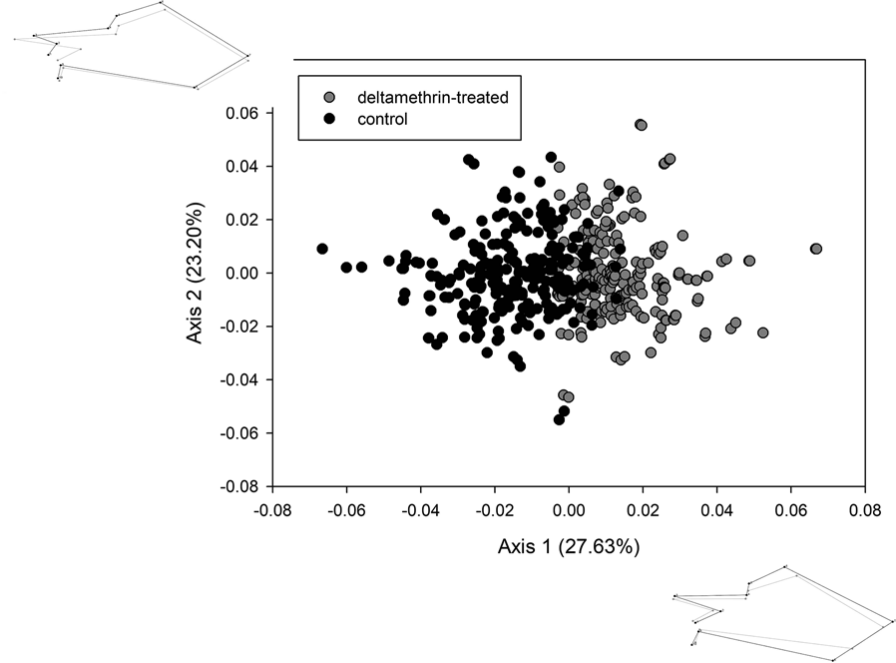
Variation in wing shape associated with the two first axes of a principal component analysis (PCA) performed on the whole dataset for female and male *T. infestans*. Black symbols indicate the control group; grey symbols indicate the deltamethrin-treated group. The changes in shape associated with the first two axes of the PCA are visualised as configurations corresponding to extreme positions of the axes. Black indicates configuration for negative extreme scores; grey indicates configuration for positive extreme scores. Shape changes correspond to an arbitrary value of 2.5 standard deviations

**Fig. 3:**
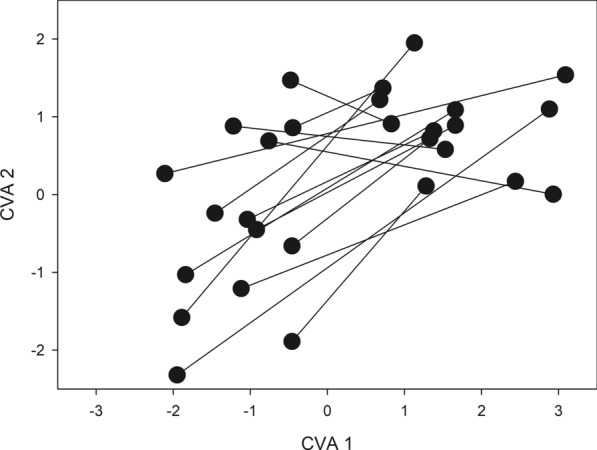
Canonical variate analysis (*CVA*) with full-sib family and treatment as factors for females and males of *T. infestans*. The symbols within the space of the first two axes represent the position of full-sib family means. Connecting lines represent the direction of shape change due to treatment within a full-sib family, i.e. shape reaction norms

For wing shape, Fig. 2 shows that individuals cluster according to treatment: the deltamethrin-treated individuals exhibit a lateral dilatation that produces wider wings. Landmarks 1, 2 and 3, positioned on the proximal part of the wings, are the least variable. The second axis corresponds to a common variation of wing shape in the two groups. The MANCOVAs (pooled sexes) showed significant effects of the terms treatment, full sib-family and the interaction between treatment and family (Table 3). Likewise for wing size, Fig. 2 suggests that: (i) wing shape reacts plastically to the insecticide treatment; (ii) genetic variation for shape is present among families; and (iii) genetic variation is present for plasticity. The CVA (including full-sib family and treatment as clustering variables) was strongly structured by treatment along the first axis, and by family along the second axis (Fig. 3). Some full-sib families exhibited roughly parallel reaction norms, but most of them were clearly different, in agreement with the interaction term in the MANCOVA.

**Table 3:** Results from multivariate analyses of covariance on the scores of the principal component analysis for wing shape under control and deltamethrin-treated conditions, full-sib families and the interaction term (treatment × full-sib family)

Effect	*df*	Pillai's test statistic	*F*	Df Num	Df Denom	Pr (> *F*)
Treatment	1	0.14	3.82	16	391	< 0.0001
Full-sib family	13	1.60	3.54	208	5239	< 0.0001
Treatment × full-sib family	13	1.32	2.85	208	5239	< 0.0001
Size	1	0.08	2.19	16	391	0.0053
Residuals	379

### Deltamethrin-treated individuals are more variable and asymmetric

**Fig. 4:**
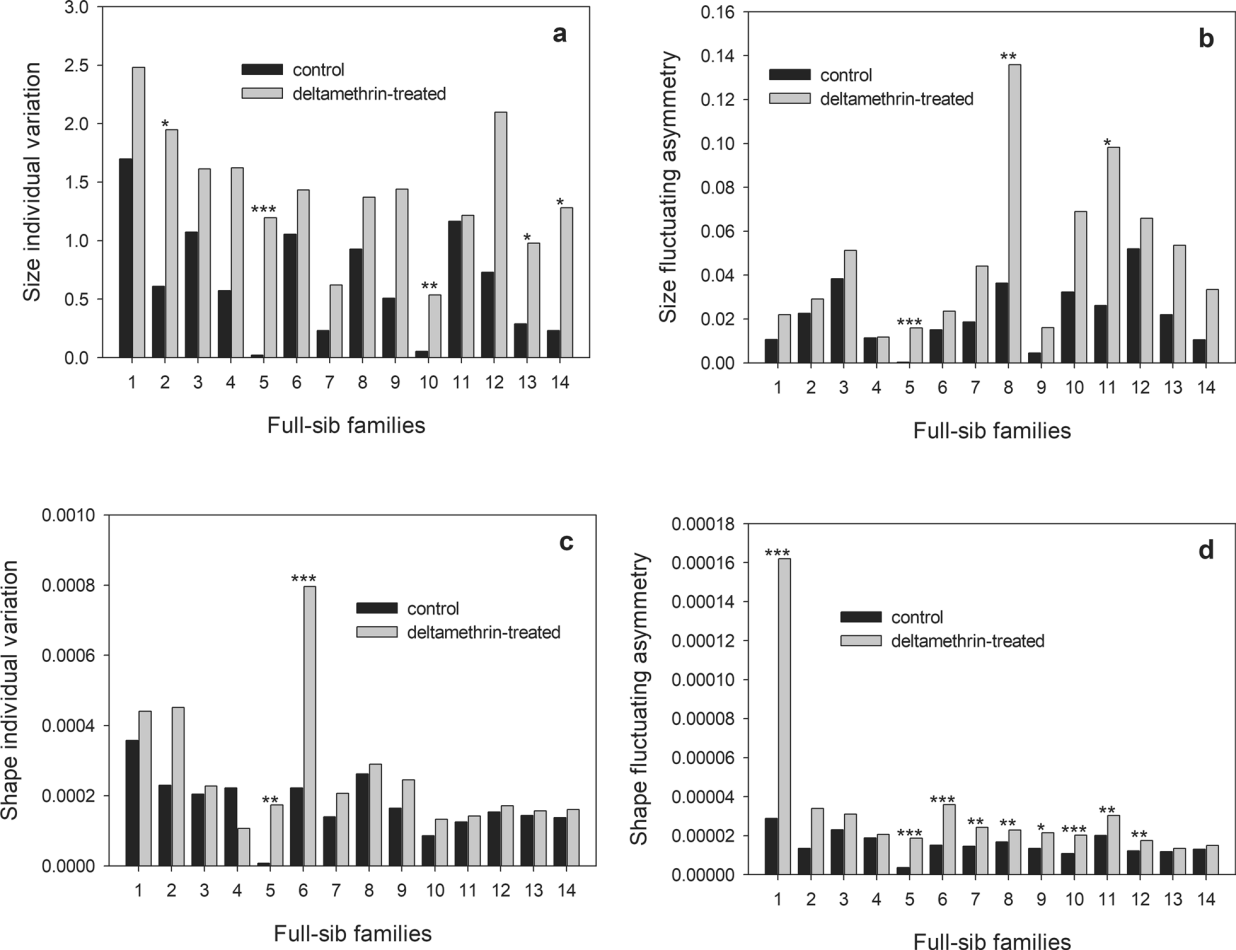
Individual and fluctuating asymmetry variation for wing size (**a**, **c**) and shape (**b**, **d**) in *T. infestans*. Each graph shows the values corresponding to control and deltamethrin-treated individuals within each full-sib family. Stars indicate the full sib-families whose variation between control and deltamethrin-treated groups showed significant differences at ***P < 0.001;,**P < 0.01 and *P < 0.05 (F-test)

Remarkably, individual variation and FA of both wing size and shape were systematically greater in deltamethrin-treated insects than in control ones in all families, with the exception of one family for shape individual variation (Fig. 4). Although systematic, these differences were not always statistically significant [i.e. for individual variation, in only 5 and 2 families for wing size and shape, respectively (Fig. 4a, c); for FA, in only 3 and 9 full-sib families for wing and shape, respectively [Fig. 4b, d]).

The Kendall tau correlation coefficient of individual size variation across full-sib families between treatments was significant (Kendall’ tau = 0.494; *P* = 0.016). The same tendency was observed for individual shape variation (Kendall’s tau = 0.486;* P* = 0.018). These results mean that the most variable families under control conditions also tended to be the most variable when treated. For FA, the Kendall tau correlation coefficient across full-sib families was also significant both for size (Kendall’s tau = 0.626; *P* = 0.002) and shape (Kendall’s tau = 0.552; *P* = 0.007). These results imply that full-sib families with the highest FA under control conditions also had the highest FA under treated conditions for both wing size and shape.

For the association between individual variation and FA in control and deltamethrin-treated conditions, the Kendall tau correlation coefficient showed that the most variable full-sib family was not the most asymmetric one for wing size (Kendall’s tau = 0.25; *P* = 0.22; Kendall’s tau = − 0.23; *P* = 0.27, for control and deltamethrin-treated conditions, respectively). For wing shape, this association was significant for both control and deltamethrin-treated conditions (Kendall’s tau = 0.50; *P* = 0.015; Kendall’s tau = 0.52; *P* = 0.011, respectively).

The effect of deltamethrin on genetic variation for wing size for both treatments was computed as the mean squares (MS) in an ANOVA on size with full-sib family as a single effect. Results of this ANOVA showed that the MS related to full-sib family effect was higher in the treated group than in the control group (MS = 1.65, *F*_size (13, 13)_ = 2.66, *P* < 0.0014), suggesting that genetic variation for wing size was increased by exposure to deltamethrin.

For wing shape, the effect of deltamethrin on genetic variation was assessed* via* the corresponding Procrustes MS. Results of this ANOVA showed the same tendency as for size: MS related to full-sib family was stronger in treated individuals (MS = 3.46, *F*_shape (224, 224)_ = 6.81, *P* < 0.001), again suggesting that deltamethrin treatment strongly increased genetic variation.

### Individual variation, FA and genetic variation exhibited different patterns of shape variation

**Fig. 5:**
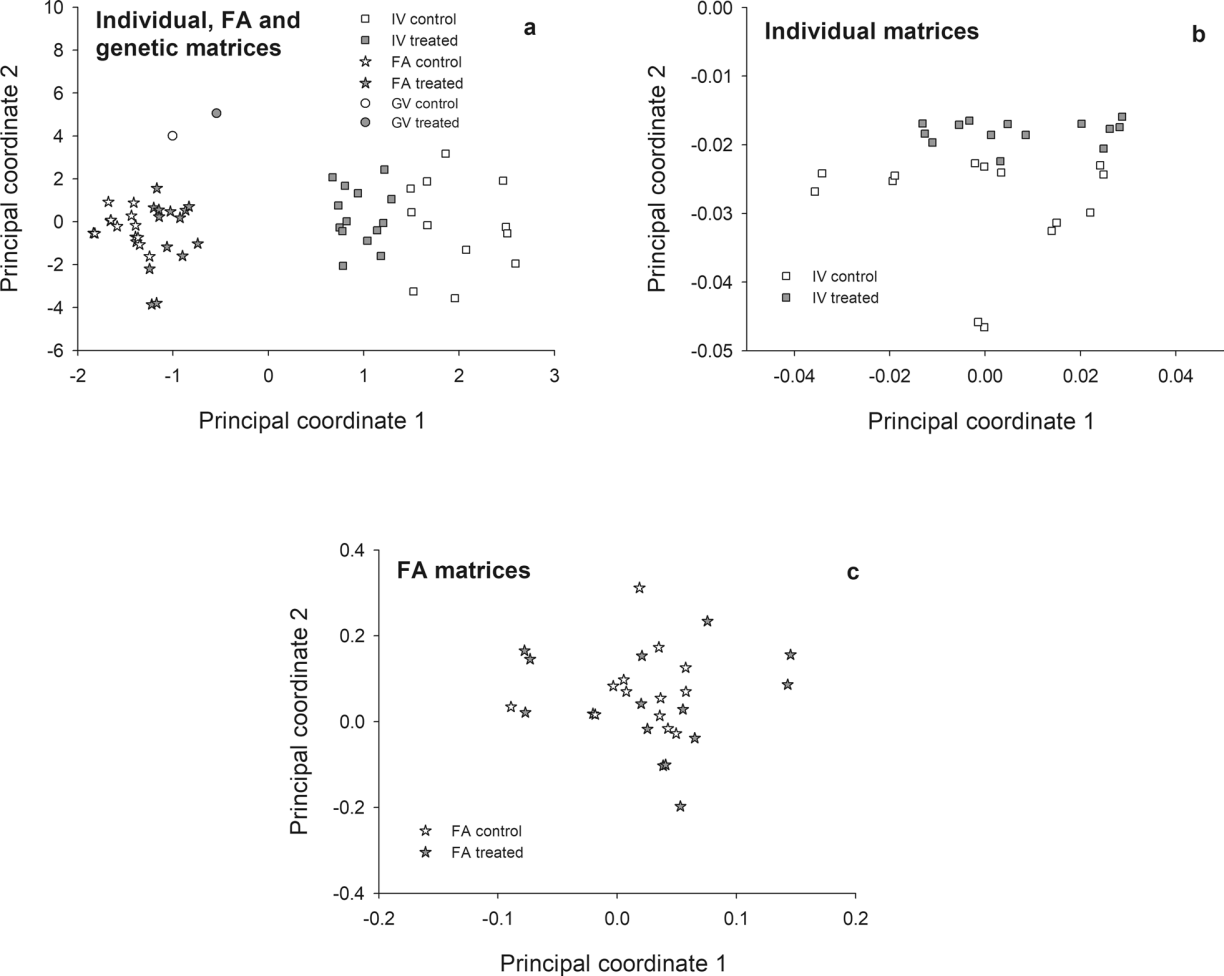
Principal coordinates analyses of the shape matrices of individual, FA and genetic variation joined together (**a**) and separately (**b**, **c**) in control and deltamethrin-treated *T. infestans*. Each symbol represents a single matrix (i.e. a full-sib family under a given treatment).* IV* Individual variation,* FA* fluctuating asymmetry variation,* GV* genetic variation

Results of the PCO applied simultaneously to individual, FA and genetic matrices showed that for both the control and treatment conditions, matrices tended to cluster according to the type of variation involved (i.e. individual, FA and genetic variation) (Fig. 5a). This clustering indicates that the patterns of shape variation among families and within and between individuals are different, suggesting that the related buffering processes are also different. When focusing on FA matrices only, no evidence of structuring was found (Fig. 5b). Deltamethrin exposure therefore did not alter the structure of FA matrices. In contrast, individual variation matrices were clustered according to treatment (Fig. 5c). Such a limitation of treatment effects to individual variation matrices (and not FA) may indicate that the processes involved in the buffering of variation within and between individuals are not similar (Fig. 5b, c).

## Discussion

Variation in genetic and environmental factors determines that individual susceptibility to insecticides is randomly distributed among individuals of a population [51]. When the population is exposed to an insecticide at a dose that does not kill all exposed individuals, the toxicant can act as a selective factor operating on the genetic variation of that distribution and promoting the evolution of resistance. The consequence of this is a shift in the dose–response curve towards higher doses of insecticide [13, 51]. The surviving individuals could present various phenotypic modifications due to exposure to sublethal doses of the insecticide, i.e. sublethal effects. In the laboratory study presented here, of 352 nymphs (belonging to 25 full-sib families) treated with a sublethal dose of deltamethrin, 172 (48%) died within 24 h. This study evaluates the response of survivors to a sublethal dose application.

Our experiments produced evidence that sublethal exposure to deltamethrin induced a plastic response of both wing size and shape. Full-sib families differed in wing size and shape, suggesting a dissimilar sensitivity to the insecticide among families. Whether changes in wing size and shape due to phenotypic plasticity affect the flight capacity of *T. infestans* (through links between flight aerodynamics and wing shape; see, for example, [26]) remains unknown. When first-instar nymphs were treated topically with a sublethal dose of deltamethrin, the size and shape of wings of the emerging adults were more variable than in adult triatomines treated with acetone only as nymphs. This result suggests an effect of insecticide exposure on developmental buffering processes. Triatomines exposed to a sublethal dose of pyrethroids exhibited bigger wings. This result may be interpreted as a selective process associated with insect body size, since it may be expected that those insects that did not survive to a sublethal dose application were smaller. For *T. infestans*, larger wings might result from natural selection which favours phenotypes adapted to insecticide exposure [52]. In previous studies, both female and male *T. infestans* collected after house spraying with pyrethroid insecticide in a context of moderate resistance had significantly larger wings than their pre-spraying counterparts [53], and wing size was positively and significantly correlated with total body length [54], which correlated closely with other metrics of body size (e.g. total body weight) [55]. These results suggest that those insects which survived an insecticide application were subjected to a selection process that favoured the survival of bigger individuals.

Within each full-sib family, individual and FA variation increased in the deltamethrin-treated group, suggesting that environmental canalisation and developmental stability were jointly impaired by insecticide exposure. Moreover, there was evidence of concordance between FA and individual variation among full-sib families for wing shape but not wing size, suggesting that the two components of developmental homeostasis (similarly affected by the treatment) were also coupled genetically. In the context of chemical control actions in the field, triatomines that survive an insecticide application in an environment without recent history of pyrethroid applications may display a highly variable population for a given trait. Insecticide-induced dose–response experiments have demonstrated that, in some cases, low doses of insecticide stimulated biological processes and increased insect survival and reproduction [56–58]. Sublethal exposures may also decrease development rates and reproduction [57] and impact on foraging ability [59].

Variation among triatomines within a treatment and full-sib family may mostly reflect microenvironmental differences (i.e. lack of environmental canalisation). Our results (most full-sib families exhibited higher levels of individual variation in size and shape relative to control individuals) clearly suggest that insecticide treatment impaired environmental canalisation. The experimental design allowed us to assess the effects of deltamethrin exposure on genetic variation by comparing variation across full-sib families under both environmental conditions. Because variation increased among deltamethrin-treated full-sib families in comparison with control full-sib families, deltamethrin application apparently also impaired genetic canalisation. This result suggests that there is a combined effect on environmental and genetic canalisation since both were higher in the deltamethrin-treated group (see, for example, [60]; but also see [61] for a discussion). This result should nevertheless be considered with caution because the variation based on 14 full-sib families does not provide a very reliable estimator of genetic variation.

The PCO ordination showed that individual, FA and genetic variation matrices were clustered separately, indicating that the patterns of shape variation within and among individuals were different. The FA and individual variation matrices were also differently affected by pyrethroid exposure: while no systematic effect on FA matrices was detected, individual matrices clustered according to treatment group, suggesting that the patterns of individual variation were affected by the treatment. This differential effect upon the two types of variation indicates that the processes involved in the buffering of intra- and inter-individual variation are not completely similar, in agreement with most previous studies (see [16] for a review).

This analysis also suggests that, in addition to the potential selective pressures that the insecticide generates, it also alters the main direction of available phenotypic variation (at least for those documented from wing shape). Genetic variation, as assessed by the among-family variance, seemed to be affected by the insecticide treatment, and might also affect the potential for further evolution.

The results of this study demonstrated that insects surviving a sublethal dose of deltamethrin showed modifications in wing size and shape, as well as in their variability. These modifications may be a direct effect of the insecticide on wing development, or they may be a pleiotropic effect of the genes that determine a low susceptibility to the insecticide of the surviving individuals [13, 38, 62]. Resistance to insecticide in the sheep blowfly *Lucila cuprina* was indeed associated with an increased FA [63]. Future studies using doses that allow the survival of all exposed insects may help determine the mechanisms underlying the sublethal effects. Moreover, quantifying the levels of molecular variation within and between full-sib families should improve our understanding of the congruence between phenotypic and molecular variation as a result of exposure to a sublethal insecticide dose.

The non-uniform application of the insecticide (due to the intricate construction features and type of materials used in domestic or peridomestic sites, such as chicken coops, goat and pig corrals), combined with the rapid degradation of pyrethroid molecules or their wash-out by sunlight, wind, dust or rain, increase the chances that triatomines are often exposed to sublethal doses of pyrethroids [64, 65]. Hence, the amount of available insecticide decays rapidly, thereby allowing the survival of less susceptible insects and the evolution of resistance. The observed morphological modifications in wing size and shape in triatomines exposed to a sublethal dose of pyrethroids may impinge on flight performance as well as on wing size. In field populations of *T. infestans*, wing size was found to be significantly and positively correlated with total body length (for example, see [54]), which correlated positively with total body weight, nymphal blood meal contents and female fecundity [55]. Thus, a single sublethal exposure to pyrethroids early in life modified adult morphological traits, which in turn may affect both the dynamics of house invasion and reinfestation and, consequently, the effectiveness of triatomine control operations.

## Conclusions

This study shows that a sublethal exposure to deltamethrin affected wing size and shape and their variability in *T. infestans*. Adult triatomines that had previously been exposed to deltamethrin had bigger wings, and both their wing size and wing shape were more variable than in those in control insects. Genetic variation, individual variation and FA jointly increased in the treated group, pointing to pyrethroid effects upon developmental, genetic and environmental canalisation. The associated patterns of wing shape variation nevertheless differed within and between individuals, suggesting that these components of developmental buffering also differed in part. By increasing genetic variation in treated populations, pyrethroid exposure might increase their adaptive potential.

## Data Availability

Data supporting the conclusions of this article are included within the article. Raw data are available from the corresponding author on reasonable request.

## Abbreviations

CS: Centroid size
FA: Fluctuating asymmetry
